# Safety and efficacy of a novel cephalomedullary nail femoral shaft fractures: a retrospective observational cohort in 33 patients

**DOI:** 10.1186/s13037-020-00269-z

**Published:** 2020-12-05

**Authors:** Jorge C. De Leon, Cooper B. Tye, Connor S. Breinholt, Khang H. Dang, Ravi A. Karia

**Affiliations:** 1 Department of Orthopaedics, UT Health San Antonio, 7703 Floyd Curl Dr, MC-7774, San Antonio, TX 78229 USA; 2 Long School of Medicine, UT Health San Antonio, San Antonio, USA

**Keywords:** META-TAN, Intramedullary nail failure, Femur shaft nonunion, Femur shaft implant failure, Femur fracture

## Abstract

**Background:**

Despite advances in femoral shaft fracture fixation, the nonunion rate remains relatively high; and there is limited data on the efficacy and failure rate of specific implants. A novel cephalomedullary nail provides the ability to treat femur shaft fractures in isolation, with associated ipsilateral femur injuries, and provides various options for proximal and distal fixation exists on the market; but literature remains limited on the safety and efficacy of this implant. The aim of this study is to evaluate the early failure rate of this cephalomedullary nail, while comparing the nonunion rate to what is currently presented in the literature. This study is the first of its kind in evaluation of a specific implant for treatment of femoral shaft fractures and ipsilateral pathology.

**Methods:**

Patients over 18 years of age, with traumatic femur shaft fractures, treated with this particular cephalomedullary nail and available for a minimum of 3-month follow-up were included for analysis. Data was collected by retrospective chart review and review of existing radiographs. Demographic data, injury details, AO/OTA fracture classification, and implant details were recorded for each patient. Primary outcome measured was implant failures (screw or nail breakage). Secondary outcomes measured included malunion, nonunion, deep infection, post-operative complications, and need for reoperation.

**Results:**

Of the 33 patients included for analysis, 1 patient went on to non-union. There were no cases of implant failure. The single nonunion was a high-energy mechanism, open fracture, and higher level AO/OTA classification. The remaining 32 reached radiographic union at 3 months.

**Conclusion:**

The nonunion rate of this novel cephalomedullary nail is comparable to what is reported in the literature. This nail is a safe and effective implant to treat femoral shaft fractures with a variety of ipsilateral femoral shaft injuries and reliably leads fracture union. Further studies are needed analyzing implant failure and comparing specific implants.

## Background

Femoral shaft fractures represent a common orthopaedic problem that may occur as the result of high-energy mechanisms in younger patients or low-energy injuries in elderly patients. Although intramedullary nailing remains the gold standard for femoral shaft fracture fixation [1–4], the question still remains for patients with ipsilateral femoral neck and shaft fractures, femoral bow variations, distal third femoral shaft fractures, number of interlocking screws, and type of proximal fixation. While the union rate and functional outcomes remain high with this technique, the nonunion rate remains 1–11% [3, 4, 6, 8, 9, 11, 12] and implant failures are severely underreported.

A novel cephalomedullary nail (Smith and Nephew Inc. TRIGEN META-TAN London, UK) is available on the market and is able to treat various femoral pathologies, such as ipsilateral femoral shaft and neck fractures, proximal femur fractures with narrow intramedullary canals, nonunion, malunion, pathologic fractures, and other complex pathology. The smaller diameter proximal integrated screws combined with smaller diameter proximal nail can provide linear compression and rotation control of the fracture site, while minimizing bone loss compared to other lag screw designs. For mid-shaft femoral fractures, the implant can be set up to accommodate better intertrochanteric screws fixation and to adjust its bow with increasing length. For distal femoral fractures, there are three screw options within 40 mm of the distal aspect, with the most proximal screw allowing for 5 mm of dynamization.

Currently, there are no studies on the outcomes or failure rate of specific implants for treatment of femur shaft fractures. There is literature on treatment of hip fractures in the elderly with specific implants, but the implant failure rate remains limited. The goal of our study is to further evaluate the safety and efficacy of this novel cephalomedullary nail in a variety of femur shaft fractures in orthopaedic trauma. We hypothesize that the union rate is comparable to what is reported in the literature for femoral shaft fractures treated with an intramedullary nail and that the implant failure rate will be low.

## Methods

A retrospective chart review was performed at an urban university-based level 1 and urban level 3 trauma centers. Study data was collected through retrospective chart review and review of the existing radiographic studies. Patients were identified through the coding database of our institution. Institutional Review Board (IRB) approval was obtained from our institution (Protocol #HSC2019328E).

Patients over 18 years of age who underwent intramedullary nail fixation of their femur shaft fractures with this particular cephalomedullary nail from January 2015–June 2019 were included in this investigation. We also included acute femur shaft fractures with ipsilateral proximal femur pathology (femoral head, neck, and intertrochanteric region), if they were treated with a single implant. Exclusion criteria included other nailing systems, retrograde nail fixation, femoral shaft fractures with an ipsilateral proximal femur fracture treated with two implants, intra-articular distal femur fracture, and pathologic fractures from neoplastic disease.

The surgical technique was according to widely established recommendations as described in the surgical guide for this cephalomedullary nail (Smith and Nephew Inc. TRIGEN META-TAN London, UK) [5]. The nail allows for adaptability with optional intertrochanteric and cephalomedullary fixation of the femur as well as three optional distal interlocking screws for proximal, midshaft, and distal third shaft fractures [1]. The implant comes in sizes 9 to 13 mm in diameter, a 14 mm proximal diameter, and interlocking options within 15 mm, 25 mm, and 35 mm of the tip of the implant with up to 15 degrees off of axis fixation. The device can also be statically or dynamically locked distally. Locking of the set screw proximally for fixed angle fixation along with the integrated lag and compression screws makes it suitable for a femoral neck and shaft fracture with an 8 mm diameter proximal lag screw to minimize risk of damage to femoral neck blood supply [1]. Regarding our post-operative protocol, the weight-bearing status of the injured lower extremity was determined by the treating surgeon. Patients were considered as incomplete follow-up if clinical and radiographic outcome data was not available for a minimum of 3 months after surgery. A minimum follow-up of 3 months was chosen since literature has reported a high rate of union at that time point [3].

The following preoperative and perioperative data points were collected from chart review and existing radiographs: age, gender, race, ethnicity, body mass index (BMI), American Society of Anesthesiologists (ASA) scale, medical co-morbidities, social history (tobacco, ethanol, illicit drug use), baseline ambulatory status (no assistive device, cane, walker, wheelchair), mechanism of injury, open or closed injury, fracture location, fracture type according to the AO/OTA classification system, operative time, nail size (as defined by diameter), number of distal screws, type of proximal fixation, primary use of bone graft, estimated blood loss, perioperative complications, and perioperative mortality.

The primary outcome measure was mechanical hardware failure. The following secondary outcome measures were recorded: malunion (defined as 5 degrees of radiographic varus/valgus malalignment, 10 degrees of radiographic procurvatum/recurvatum malalignment, or more than 10 degrees of clinical rotational deformity), non-union (as defined by the need of a secondary surgical procedure to improve healing), peri-implant fracture, postoperative surgical complications, such as wound dehiscence, hematoma, superficial infection, deep infection, sepsis, and postoperative medical complications, such as thromboembolic events, pneumonia, urinary tract infection, and myocardial infarction.

## Results

Based on the current procedural technology (CPT) 25,706, a total of 435 patients were initially screened for participation in this retrospective study. However, 375 patients did not meet our inclusion criteria: 125 patients treated with a retrograde nail, 220 patients fixed with a different antegrade implant, 23 individuals under the age of 18, and 7 patients with duplicate medical record numbers. Therefore, a total of 60 patients treated with this cephalomedullary nail were investigated in this study. However, 27 of the patients did not meet the minimum 3-month follow up, but none of these patients had radiographic evidence of implant breakage or failure at the last visit. The demographic and clinical outcome data of the remaining 33 patients are represented in Tables 1, 2 and 3.

**Table 1:** Patient demographics

**Age [years]**	**Median 42.5 years (Range 18–89)**
**Tobacco Use**	
**Yes**	**10**
**No**	**23**
**Gender:**	
**Female**	**14**
**Male**	**19**
**Diabetes Mellitus:**	
**No**	**31**
**Yes**	**2**
**Body Mass Index [kg/m**^**2**^**]**	**Median: 27.2 (Range 20.6 to 60.8)**
**Obesity:**	
**Non-obese (BMI < 30.0 kg/m**^**2**^**)**	**18**
**Obese (BMI ≥ 30.0 kg/m**^**2**^**)**	**15**
**Injury Mechanism:**	
**Ground level fall**	**11**
**Fall from height**	**0**
**Motor vehicle collision**	**18**
**Bicycle accident**	**0**
**Motorcycle collision**	**1**
**Gunshot injury**	**1**
**Motor vehicle vs. Ped**	**1**
**Crushed Injury**	**0**
**Other (golf cart, ATV, jet ski)**	**1**

**Table 2:** Clinical data

**OTA/AO Fracture Classification:**	
**32-A1**	**11**
**32-A2**	**7**
**32-A3**	**5**
**32-B1**	**1**
**32-B2**	**3**
**32-B3**	**2**
**32-C1**	**0**
**32-C2**	**2**
**32-C3**	**2**
**Cephalomedullary screws**	**18**
**Intertrochanteric screw**	**15**
**Distal Screws**	
**Zero**	**1**
**One**	**8**
**Two**	**22**
**Three**	**2**
**Length of Hospital Stay [days]**	**Median 6.9 (Range: 1–75)**
**Length of Follow up [weeks]**	**Mean 12 (SD: 14.1)**
**Operative Time from Skin Incision [min]**	**Median 107 (Range: 52–225)**
**Estimated Blood Loss [mL]**	**Median 182 (Range: 75–325)**
**Weight bearing Status**	**Non-weight bearing: 7**
	**Touch-down weight bearing: 2**
	**Weight bearing as tolerated: 24**

**Table 3:** Complications

**Mechanical Hardware Failure:**	
**Screw Cutout**	**0**
**Broken Distal Screws**	**0**
**Distal Screw Loosening**	**0**
**Loose Lag Screws**	**0**
**Delayed Union**	**0**
**Postoperative Complications:**	
**Small bowel obstruction**	**1**
**Morel-Lavalee lesion**	**1**
**Retroperitoneal hematoma**	**1**
**Clavicle non-union**	**1**
**Distal Radius fracture after fall**	**1**
**Postoperative Surgical Complications:**	
**Superficial Wound Infection**	**0**
**Deep Wound Infection**	**1**
**Revision Surgery:**	
**Malrotation**	**0**
**Malunion**	**0**
**Nonunion**	**1**
**Hardware Removal:**	
**Symptomatic Hardware**	**0**
**Deep Infection**	**0**
**Loose Lag Screw**	**0**

**Fig. 1:**
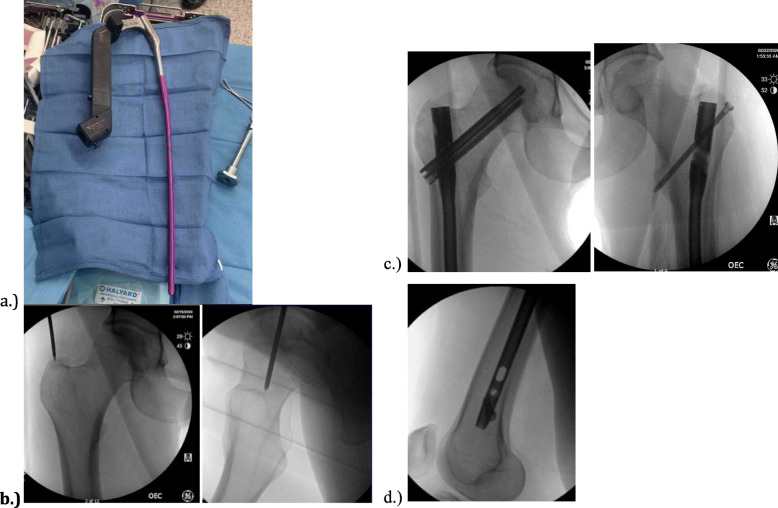
Showing in (**a**) the cephalomedullary nail assembled on the back table. The ideal starting point is shown in (**b**) on an AP and lateral image. The two types of proximal fixation are shown in (**c**). The distal interlocking options are shown in (**d**)

All 33 patients were treated with this novel cephalomedullary nail, shown in Fig. 1a, for their femur shaft fractures. All nails had a trochanteric starting point similar to that shown in Fig. 1b. The fracture patterns included eleven AO/OTA 32-A1 fractures, seven 32-A2 fractures, five 32-A3 fractures, one 32-B1 fractures, three 32-B2 fractures, two 32-B3 fractures, two 32-C2 fractures, and two 32-C3 fractures. The type of proximal fixation was cephalomedullary mode in 18 fractures and femoral mode in 15 fractures, as shown in Fig. 1c. Of the 18 patients with cephalomedullary fixation, one patient had a non-displaced ipsilateral femoral neck fracture while the remaining 17 patients were performed based on surgeon preference. All 15 of the femoral mode fixations were performed by surgeon choice as well. The number of distal interlocking screws was zero in 1 patient, one in 8 patients, two in 22 patients, and three in 2 patients. The number of distal interlocking screws was chosen by surgeon preference. The three distal interlocking options can be seen in Fig. 1d. There were no incidences of screw or nail breakage.

## Discussion

The current standard treatment for femoral shaft fracture remains the intramedullary nail due to its minimally invasiveness, allowance for early weight bearing, and minimal disruption to soft tissue [1–4]. However, the incidence of femoral shaft nonunion after intramedullary nailing is still 1–11% [3, 4, 6–9, 11, 12, 14]. This novel cephalomedullary nail is theorized to promote bone healing with its unique antegrade intramedullary design that can treat a variety of femur fracture pathology. In our retrospective cohort, we observed 1 nonunion in 33 patients without any mechanical failures, confirming our hypothesis that this nailing system is a safe and effective.

**Fig. 2:**
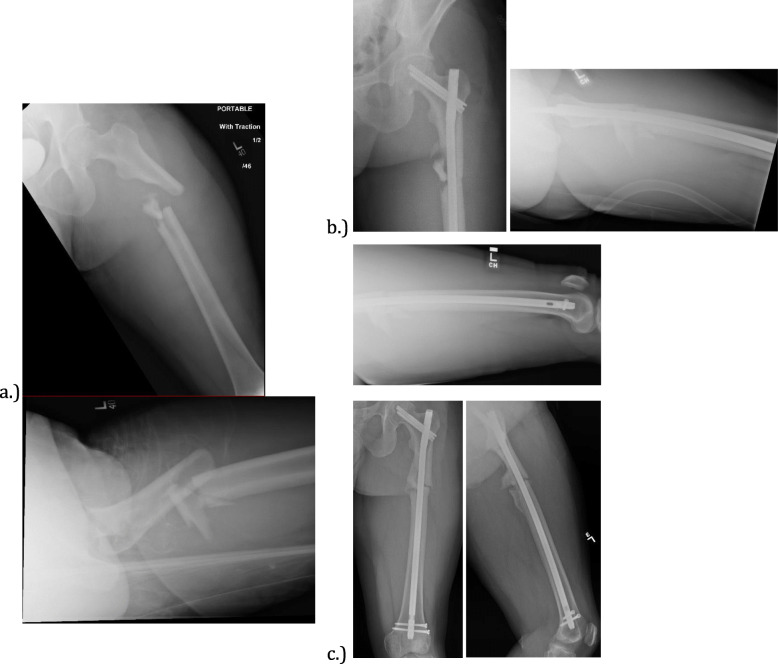
Shows an AO/OTA 32-B3 subtrochanteric femur fracture in 34 year old male in (**a**). The fracture was fixed with the antegrade cephlomedullary nail with two distal interlocking screws shown in (**b**). At the 6 month visit, the patient had a delayed union shown in (**c**) with continued pain over the fracture site and with weight bearing

The patient with a femur shaft nonunion can be further interpreted by the demographics. The patient is a 34 year old male non-smoker, involved in a high speed motor vehicle collision, who sustained an open proximal third AO/OTA 32-B3, shown in Fig. 2a, treated with a reamed, statically locked intramedullary nail with a cephalomedullary screw and two distal interlocking screws shown in Fig. 2b. Our patient did not have any immediate postoperative complications, but did have risk factors for nonunion: high-energy mechanism, comminuted fracture on the AO/OTA classification, and an open fracture. At his six-month follow up visit he was found to have persistent pain at the fracture site, limited mobility, and radiographic evidence of a delayed union shown in Fig. 2c. He ultimately went on to non-union, which was successfully treated with a reamed exchange nail augmented with autograft, and supplemental plate fixation.

**Fig. 3:**
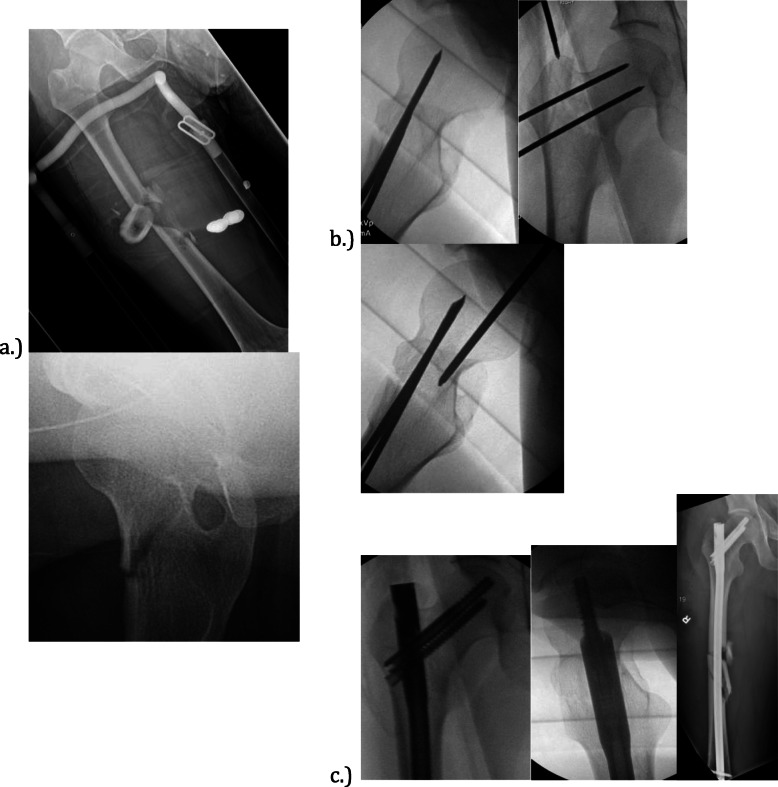
Closed comminuted right femur shaft fracture with a minimally displaced femoral neck fracture shown in (**a**). Two terminally threaded K-wires placed anterior to starting point to control rotation of the femoral head are shown in (**b**). Final images of this particular cephalomedullary nail used to treat both injuries are shown in (**c**)

The versatility of the implant can be demonstrated by the case of a 22-year old male passenger involved in a high-speed motor vehicle collision. He sustained a closed comminuted right femoral shaft fracture with a non-displaced right femoral neck fracture shown in Fig. 3a. Temporary fixation of the neck was obtained first with threaded k-wires, followed by insertion of a reamed cephalomedullary nail with cephalomedullary fixation and two distal interlocking screws shown in Fig. 3b and c. Eventual union of both fractures was obtained at 5 months.

Risk factors shown to correlate with femoral shaft nonunion are smoking, fracture reduction, AO/OTA fracture classification, un-reamed nails, open fractures, increased body mass index, and delay to weight bearing [4, 6, 8–13]. However, age, gender, direction of intramedullary nail, and number of interlocking screws has not been shown to correlate with femoral shaft nonunion [9, 10]. The Canadian Orthopaedic Society [6] reported that un-reamed intramedullary nails have a significantly higher nonunion rate in femoral shaft fractures; however, Mestsemakers et al. [11] did not find a significant relationship between unreamed nails and nonunion. Taitsman et al. [8] reported that tobacco use, open fracture, and delayed weight bearing are risk factors for nonunion after intramedullary nailing of femoral shaft fracture. In a multivariate analysis, Metsemakers et al. [11] only found AO/OTA classification as a risk factor for nonunion. Higher energy mechanisms, such as motor vehicle accidents, motorcycle collisions, and high velocity gunshot wounds, can lead to a higher occurrence of open fractures, increased periosteal stripping, and comminuted fractures, which contribute to the higher rate of nonunion.

There is a scarcity of literature on femoral shaft fractures treated with a specific intramedullary implant, especially evaluating implant failure, nonunion rates, and functional outcomes. There is also a scarcity of literature on specific implant failures rates. The versatility of this unique cephalomedullary nail lies its multiple modes of fixation with intertrochanteric and cephalomedullary screws in the proximal femur along with optional distal interlocking screws for distal fixation. Also, its cephalomedullary screws are a smaller diameter than other implants resulting in less bony purchase which can have a theorized decrease in the risk of blood supply disruption to the femoral head. This antegrade nailing system is inserted through a trochanteric entry point, which is associated with better femoral version, and lower revision rates compared to the piriformis start point [15, 16]. With antegrade nailing, elderly patients can be expected to have more functional deficits compared to their younger counterparts [16–19]. Overall, our investigation shows that this particular cephalomedullary nail has a nonunion rate for femoral shaft fractures comparable to the literature but also allows for multiple modes of fixation with a single implant. There was no incidence of implant failure, but with lacking data in the literature on this, no comparison can be drawn.

Limitations of our study include its retrospective design. Our study does not allow for conclusions on long-term outcomes and had a relatively small sample size of 33 patients. Also, we have a number of patients lost to follow up prior to 3 months, but none demonstrated signs of hardware failure at their last follow up. The configuration of nail fixation was not standardized and chosen under the discretion of the treating surgeons. A standardized protocol would be difficult given the significant variability of fracture patterns. In addition, we did not have a comparison group treated with a different nailing system.

## Conclusion

Although fixation of femoral shaft fractures has been successful with modern implants, the nonunion rate remains high in a subset of fractures and there is the potential for implant failure in a non-united fracture. Regardless of implant used tobacco use, open fractures, unreamed nails, and high-energy fracture patterns are associated with higher nonunion rates. This particular cephalomedullary nail shows similar nonunion rates as reported in the literature with a low failure rate; but allows for multiple modes of fixation in the same femur. While we showed that it is a safe and reliable implant for fixation of femoral shaft fractures, we could not overcome certain patient demographics. Further randomized studies are needed to compare different nailing systems to determine if a particular nail is superior to others.

## Data Availability

The data presented in this manuscript will NOT be shared. The data will not be shared because it is not available through a database. It is only available through search of patient electronic medical record.

## Abbreviations

TAN: Trochanteric antegrade nail
mm: Millimeters
IRB: Institutional review board
BMI: Body mass index
ASA: American Society of Anesthesiologist
AO/OTA: Arbeitsgemeinschaft für Osteosynthesefragen/Orthopaedic Trauma Assocation
CPT: Current procedural terminology
