# Correction to: Safety and efficacy of a novel cephalomedullary nail in femoral shaft fractures: a retrospective observational cohort in 33 patients

**DOI:** 10.1186/s13037-021-00292-8

**Published:** 2021-04-29

**Authors:** Jorge C. De Leon, Cooper B. Tye, Connor S. Breinholt, Khang H. Dang, Ravi A. Karia

**Affiliations:** 1 Department of Orthopaedics, UT Health San Antonio, 7703 Floyd Curl Dr,MC-7774, San Antonio, TX 78229 USA; 2 Long School of Medicine, UT HealthSan Antonio, San Antonio, USA

**Correction to: Patient Saf Surg 14, 44 (2020)**

**https://doi.org/10.1186/s13037-020-00269-z**

Following publication of the original article [1], the authors noticed that the title needs to be updated from “Safety and efficacy of a novel cephalomedullary nail femoral shaft fractures: a retrospective observational cohort in 33 patients” to “Safety and efficacy of a novel cephalomedullary nail in femoral shaft fractures: a retrospective observational cohort in 33 patients”. The original article has been corrected.
